# Editorial: Molecular pathology, biomarkers and therapeutics of brain tumor and neurodegenerative disease

**DOI:** 10.3389/fnmol.2025.1584157

**Published:** 2025-03-11

**Authors:** Junhui Wang

**Affiliations:** Lunenfeld-Tanenbaum Research Institute, Mount Sinai Hospital, Toronto, ON, Canada

**Keywords:** brain tumor, neurodegenerative diseases, biomarkers, central nervous system, glioma

Both brain tumors and neurodegenerative diseases are among the most prevalent and devastating disorders affecting the human brain. Despite significant progress in research and clinical practice over the past decade, both conditions continue to be major contributors to global morbidity and mortality. A deeper understanding of their molecular pathological features is essential, not only for uncovering the underlying mechanisms of these diseases but also for advancing the development of novel diagnostic biomarkers and therapeutic strategies.

In the field of neuro-oncology, the 2021 World Health Organization (WHO) Classification of Tumors of the Central Nervous System (CNS) introduced transformative changes, highlighting the critical role of molecular diagnostics in CNS tumor classification. This updated framework has redefined diagnostic standards, expanded the range of recognized tumor entities, and refined prognostic stratification. These advancements have enabled more accurate diagnoses and personalized treatment approaches, ultimately improving patient outcomes. Similarly, although the exact etiology and pathogenesis of neurodegenerative diseases such as Alzheimer's disease (AD) and Parkinson's disease (PD) remain incompletely understood, recent investigations have illuminated key molecular mechanisms driving disease progression. These insights have not only advanced our understanding of the pathobiology underlying neurodegeneration but also revealed promising avenues for therapeutic intervention.

This Research Topic aims to present the latest advancements in the molecular pathology of neuro-oncological and neurodegenerative disorders, with the goal of providing novel insights into their diagnosis, prognosis, and treatment. We seek to highlight groundbreaking discoveries, emerging trends, unresolved challenges, and future directions within these dynamic fields. Our scope includes a comprehensive exploration of disease pathogenesis across various dimensions, including molecular, cellular, structural, and functional aspects. We will also focus on the identification and validation of biomarkers and the development of cutting-edge technologies that hold promise for improving diagnostic accuracy, prognostic precision, and therapeutic efficacy. Given the growing application of multi-omics technologies—such as genomics, transcriptomics, proteomics, metabolomics, and epigenomics—in characterizing the molecular landscapes of human diseases, we particularly welcome contributions that focus on the multi-omics pathology of brain tumors and neurodegenerative disorders.

Such multidisciplinary approaches hold immense potential for unraveling the complexity of these diseases, identifying novel therapeutic targets, and advancing precision medicine tailored to individual patients. Through this Research Topic, we hope to foster collaborative efforts and inspire further innovation in understanding and combating these formidable brain disorders.

Astrocytoma is a brain tumor that develops from astrocytes. Although it is a rare tumor of the central nervous system, astrocytoma is the most common type of glioma. Astrocytoma often presents with non-specific clinical symptoms and lack prominent histological features, making accurate diagnosis and prompt treatment a significant challenge. Therefore, there is an urgent need to identify mutant genes to provide reliable early diagnoses and further our understanding of the etiology in order to develop effective therapies. In the first research article here, Wu et al. employed fluorescence *in situ* hybridization (FISH) and next-generation sequencing (NGS) techniques to sequence samples from four female patients with astrocytoma. They discovered that the EWSR1 gene (Exon 1–7) could fuse with BEND2 (Exon 2–14), BEND2 (Intergenic), or NUDT10 (Intergenic) in the tumor tissues of three patients. In the fourth case, an MN1 (Exon 1)-BEND2 (Exon 2) fusion was identified. More importantly, the EWSR1-NUDT10 gene fusion represents a new fusion type in astrocytoma. Using FISH or NGS, this study provides novel insights into the molecular mechanisms of astrocytoma and aids in its differential diagnosis.

Remyelination is a process that restores axonal insulation, promoting neuroprotection and recovery after myelin damage. Developing new pharmacological approaches to facilitate remyelination is crucial for treating diseases related to myelin damage, such as multiple sclerosis. In another study, Recchia et al. investigated whether budesonide, a type of glucocorticoid (GC), could promote axonal myelination and the potential underlying mechanism. They found that budesonide, a well-known GC that binds to the Smo cysteine-rich domain (CRD) and prevents Smo translocation to the cilium in fibroblasts, significantly promoted axonal myelination by treating oligodendroglial cells. This occurred through the reduction of Smo CRD conformational flexibility, leading to increased Myelin Basic Protein (MBP) expression. Additionally, they reported that budesonide exerted its beneficial effects by inhibiting Smo-mediated canonical signaling and subsequently activating the Liver Kinase B1 (LKB1)/AMP-activated protein kinase (AMPK) pathway.

Coatomer protein complex zeta 2 (COPZ2) is a subunit of coatomer protein complex I and is involved in various cancers (Geng et al.). In this Research Topic, Geng et al. investigated the potential involvement of COPZ2 in glioma by analyzing COPZ2 expression data and related clinical data from The Cancer Genome Atlas (TCGA). Multiple analyses were used to assess the relationship between COPZ2 and other prognostic factors in glioma, as well as to predict the potential biological mechanisms involved. Their data indicated that COPZ2 expression was high in glioma, and its expression was associated with age and WHO grades, acting as an unfavorable factor for poor glioma prognosis. In *in vitro* studies, they found that silencing COPZ2 significantly inhibited the malignant behaviors of glioblastoma cells by regulating the PI3K-AKT signaling pathway. In conclusion, while associated with the prognosis and progression of glioma, COPZ2 could serve as a potential diagnostic and prognostic biomarker for glioma.

Alzheimer's disease (AD) is a neurodegenerative disorder that leads to a progressive decline in memory, thinking, and behavior. Biomarkers play a crucial role in diagnosing, monitoring, and understanding diseases such as AD. Fang et al. investigated the potential of using serum neurofilament light chain (NfL) and glial fibrillary acidic protein (GFAP) for the early diagnosis of AD and for distinguishing between AD and mild cognitive impairment (MCI). They collected venous blood samples from patients in three groups: healthy controls (HC), MCI, and AD. Their results confirmed that both serum NfL and serum GFAP levels could independently diagnose AD as biomarkers, as significant differences in the expression levels of these two proteins were observed in the serum of the AD and MCI groups compared to the HC group. However, among the MCI and AD groups, significant differences were only found in GFAP (*p* < 0.01), while no difference in NfL levels was observed between the two groups.

Identifying new biomarkers is essential for developing more precise and personalized treatments for gliomas. Yin et al. utilized differential expression analysis and Cox regression analysis to identify key survival-related genes (SRGs) within glioma datasets, aiming to develop a prognostic risk model to predict patient outcomes. This approach facilitates the prompt stratification of glioma patients based on their prognosis, providing valuable insights into potential therapeutic targets. They successfully identified vesicle-associated membrane protein 2 (VAMP2) and vesicle-associated membrane protein 5 (VAMP5) as two SRGs that could influence the prognoses of glioma patients. This novel risk model, which was further validated through bioinformatics analysis and experimental assays, may prove useful in clinically predicting the response of glioma patients to immunotherapy.

In conclusion, the studies presented in this Research Topic provide valuable insights into the application of biomarkers in brain tumors and neurodegenerative diseases. These findings emphasize the critical role of biomarkers in the early diagnosis and treatment of these conditions. Overall, our Research Topic underscores the urgent need for continued research to identify new biomarkers in brain diseases, utilizing a variety of innovative tools and technologies.

